# Scents of right and wrong: how odor-induced affect shapes moral judgment in adults and preschoolers

**DOI:** 10.3389/fpsyg.2026.1834581

**Published:** 2026-07-07

**Authors:** Weiwei Li, Yuning Zhang, Ziqing Luo, Qingqing Cao, Xihua Zeng

**Affiliations:** 1 College of Education (Normal School), Dongguan University of Technology, Dongguan, China; 2 Guangdong University of Education, Guangzhou, China; 3 Department of Psychology, School of Public Health, Southern Medical University, Guangzhou, China; 4 Department of Psychiatry, Zhujiang Hospital, Southern Medical University, Guangzhou, China; 5 Shenzhen Longhua District Shangjun Kindergarten, Shenzhen, China; 6 The Affiliated Preschool of Shenzhen Longhua District Education Institute Early Childhood Education Group, Shenzhen, China

**Keywords:** domain specificity, early childhood, moral development, moral judgment, olfactory affect

## Abstract

Recent research suggests that incidental affect, including odor-elicited emotion, can shape moral judgment in adults. However, little is known about how this influence develops across early childhood. The present research examined whether pleasant, neutral, and unpleasant odors modulate moral evaluations of care and purity violations in adults and preschool-aged children. In Study 1, 36 undergraduates completed a within-subjects task under three odor conditions. In Study 2, 93 children aged 3 to 6 years completed a developmentally adapted version of the same task. Among adults, odor effects were domain-selective: unpleasant odor increased condemnation relative to pleasant odor in the care domain, whereas purity judgments were unaffected. Among children, analyses by age group indicated that odor effects were confined to the 4- to 5-year-old group. Within this subgroup, unpleasant odor was associated with higher wrongness ratings than neutral odor in both domains, although the overall pattern differed across domains. Taken together, these findings suggest that the influence of incidental odor on moral judgment may shift from a relatively broad pattern in early childhood to a more domain-selective pattern in adulthood. This developmental change is consistent with increasing cognitive control and the progressive differentiation of moral reasoning across domains.

## Introduction

Incidental emotions, affective states unrelated to the event under evaluation, can shape moral judgment. Adults exposed to disgust-inducing stimuli, such as unpleasant odors, bitter tastes, or aversive images, increase the severity of moral condemnation (Eskine et al., 2011; Schnall et al., 2008b; Seidel and Prinz, 2013). Moral judgment, however, does not work the same way across all situations. It varies across distinct content domains that recruit different cognitive and affective processes. A critical question therefore remains open: does incidental affect intensify condemnation across all moral domains equally, or does it act selectively on those domains theoretically linked to specific emotions?

Current theories of moral cognition identify several content areas. Among them, two domains provide a particularly informative contrast for testing whether incidental emotions work selectively or broadly, because they are theoretically linked to distinct intuitive emotions. Care morality concerns evaluations of harm, suffering, and failures of compassion, and is typically grounded in concrete, interpersonally salient events (e.g., witnessing someone step on a kitten’s tail; Haidt and Joseph, 2004). Purity morality, by contrast, involves concerns about contamination, bodily integrity, and symbolic transgression (e.g., witnessing someone drink urine; Graham et al., 2013; Haidt, 2001). These two domains are theoretically and empirically dissociable, and prominent accounts argue that they recruit distinct intuitive emotions: care violations evoke empathic concern about harm, while purity violations trigger disgust (Haidt and Joseph, 2004; Horberg et al., 2009). If the coupling between moral domain and emotion is domain-specific, incidental disgust from odors should selectively increase purity judgments; if the coupling is domain-general, odor effects should extend to care judgments or even predominate there. Testing between these possibilities requires studies that manipulate both emotional input and moral content type. A second important question is whether such effects appear as soon as children can make moral judgments, or only develop with cognitive growth. Existing research has studied almost exclusively adults, leaving the developmental pattern largely unknown. The present research addresses both questions by examining how pleasant, neutral, and unpleasant odors influence moral evaluations of care and purity violations in early childhood and adulthood.

### Olfactory influences on moral judgment

Olfaction is one of the most evolutionarily ancient human senses, and a substantial body of research demonstrates that odors profoundly shape human emotion, cognition, and behavior. Pleasant fragrances facilitate positive mood, prosocial behavior, and evaluative leniency, whereas unpleasant odors elicit aversion and negativity (Baron and Thomley, 1994; Chen and Haviland-Jones, 2000; Hermans et al., 1998; Holland et al., 2005; Liljenquist et al., 2010). These effects reflect the distinctive neuroanatomy of the olfactory system. Unlike other senses, olfactory input reaches affective brain regions, including the amygdala and orbitofrontal cortex, through direct projections that bypass extensive cortical relay (Boesveldt et al., 2010; Gottfried et al., 2002; Rolls et al., 2003). Neuroimaging studies have further shown that pleasant and unpleasant odors activate dissociable circuits: pleasant odors primarily engage the medial orbitofrontal cortex, a region tied to pleasure and reward, whereas unpleasant odors engage the anterior insula and adjacent areas linked to disgust and aversion (Bensafi et al., 2002; Rolls et al., 2003; Seubert et al., 2010). This rapid and largely unconscious affective processing offers a concrete neural basis for understanding how olfactory stimuli may shape evaluative processes, including moral judgment.

Two complementary frameworks explain how this influence occurs. The affect-as-information account (Schwarz and Clore, 1983) proposes that people sometimes misattribute their current emotional state to the object they are evaluating, especially when the source of the emotion is ambiguous or goes unnoticed. Because the olfactory pathway generates affect rapidly and without requiring explicit source attribution, odor-induced emotion can merge with the response to scenario content, making moral condemnation feel either more or less severe. The embodied cognition framework offers a broader perspective, arguing that cognitive processes are grounded in bodily states and sensorimotor experience (Barsalou, 2008; Wilson, 2002), and that emotional states in particular shape evaluative cognition through their bodily substrates (Niedenthal, 2007). Olfactory affect therefore does not merely provide evaluative information; it constitutes part of the bodily foundation from which moral responses emerge.

Studies guided by these frameworks consistently show that odors shape adults’ moral judgments. Pleasant odors tend to increase prosocial behavior and make people more lenient in their evaluations, whereas unpleasant odors, particularly those that elicit disgust, are associated with harsher moral condemnation (Cecchetto et al., 2019; Holland et al., 2005; Liljenquist et al., 2010; Schnall et al., 2008b). What remains contested is whether these effects are domain-specific or domain-general. Meta-analytic evidence indicates that incidental disgust effects on moral judgment are small and inconsistent across studies (Landy and Goodwin, 2015). One reason for this inconsistency may be methodological: most prior studies have examined a single moral domain at a time, making it difficult to determine whether odor effects are confined to specific content (e.g., purity) or extend across moral evaluations more broadly. Three theoretical frameworks offer competing accounts of these mixed findings.

### Three competing theoretical frameworks

These competing accounts, namely Moral Foundations Theory, the olfactory evolutionary hypothesis, and dual-process models, make distinct, empirically separable predictions about (a) the scope of olfactory influence across moral domains and (b) its developmental trajectory. Because existing studies have rarely crossed odor valence, moral domain, and age within a single design, these frameworks have remained largely indistinguishable on empirical grounds. The present research crosses odor valence with moral domain in both adults and preschoolers, creating the conditions under which the three frameworks generate distinguishable patterns of predictions across the age × domain × valence space, although their predictions for any single condition may overlap.

Moral Foundations Theory (MFT) offers the most domain-specific prediction of the three frameworks. According to MFT, distinct emotions evolved to regulate distinct categories of moral violation, with disgust tightly coupled to concerns about purity and contamination rather than harm or care (Haidt, 2001; Haidt and Joseph, 2004, 2008). On this account, unpleasant odors should selectively intensify condemnation of purity violations relative to care violations, with little or no effect in care judgments (Liuzza et al., 2019; Schnall et al., 2008a; Schnall et al., 2008b). Empirical support for this prediction comes from Horberg et al. (2009). In their Study 3, disgust evoked through film clips specifically intensified judgments of purity transgressions while leaving care and justice evaluations largely unaffected. Additional findings confirm that disgust cues amplify moral condemnation in the purity domain but not necessarily in the care domain (Liuzza et al., 2019; Schnall et al., 2008a). MFT itself does not specify when the disgust–purity coupling develops, but Rozin and colleagues argue that disgust is a “delayed-maturation” emotion whose moral applications develop gradually throughout childhood (Rozin et al., 2000). This creates two possible developmental predictions from MFT: under a strict interpretation, the disgust–purity coupling reflects innate moral architecture and should appear as soon as children can make moral judgments; under a Rozin-influenced interpretation, selective purity effects should emerge only after disgust has matured and should therefore be stronger in adults than in preschoolers.

The olfactory evolutionary hypothesis adopts a broader functional perspective. Olfaction evolved as a general-purpose system for detecting environmental threats and guiding adaptive behavior (Wilson, 2002). Foul odors such as cadaverine trigger avoidance and psychological defense, while pleasant smells promote approach behavior (Barnett et al., 2022). On this view, affect signals stimulus relevance and coordinates physiological and behavioral responses to adaptive challenges; behavior biasing emerges as one consequence of these broader functions rather than as the sole evolutionary purpose of affect. Strong negative affective states such as disgust and aversion may therefore broadly heighten evaluative vigilance, including moral condemnation, across diverse content domains rather than being yoked to a single domain (Homan et al., 2017; Wu, 2013). Consistent with this functional view, Wu (2013) reported that exposure to unpleasant odors elevated condemnation in both purity- and harm-relevant scenarios, suggesting that olfactory disgust may operate as a general affective amplifier of moral severity rather than a purity-specific cue. Because the proposed mechanism does not depend on domain-specific cognitive architecture, this framework predicts broad, undifferentiated odor effects across the lifespan, beginning at the earliest age at which children reliably categorize odors as pleasant or unpleasant.

A third perspective comes from dual-process models of moral judgment, which emphasize the interaction between intuitive emotional responses and controlled cognitive processes (Greene et al., 2004; Greene and Haidt, 2002). Rather than treating emotional influence as inherently general or domain-specific, this framework holds that the impact of incidental emotion depends on how much each judgment recruits rapid affective appraisal versus deliberative reasoning. Care violations typically depict concrete and emotionally vivid harm, and judgments of such acts rely heavily on rapid affective appraisal (Greene et al., 2001). They are therefore well positioned to be amplified by co-occurring negative affect from an extraneous source. Purity dilemmas, by contrast, often involve acquired norms about hygiene and bodily integrity that can engage rule-based evaluation alongside intuitive disgust (Greene, 2007); to the extent that controlled processes participate, they may attenuate the impact of incidental affect. The dual-process framework also makes an explicit developmental prediction. Because executive control, source monitoring, and the ability to discount task-irrelevant affect develop gradually across the preschool and school years (Smetana et al., 2012; Wainryb et al., 2005), young children should show broader, less domain-differentiated effects of odor on moral judgment, with selectivity emerging only as cognitive control matures.

### Olfactory development and moral cognition in early childhood

These three frameworks share a focus on adult cognition, yet each rests on implicit or explicit assumptions about the affective and regulatory resources that give rise to the predicted effects. Early childhood therefore offers a critical test of these accounts, because preschoolers’ affective and regulatory systems are still developing and lack some of the resources that adult-focused accounts presuppose. We therefore turn to what is known about the development of olfactory affect and moral cognition during the preschool years.

Although basic olfactory abilities emerge early in life, the interpretation and regulation of affective responses continue to develop throughout childhood (Schaal, 1988; Sullivan et al., 1991). By preschool age, children can detect and discriminate odors at levels comparable to those of adults (Cain et al., 1995; Hummel et al., 2007). Yet the capacity to interpret, categorize, and assign social meaning to odors is still emerging during this period, shaped by both cognitive development and accumulating social experience (Sorokowska et al., 2015). Children’s affective responses to odors are also less differentiated than adults’. Cavazzana et al. (2018) found that the youngest children (younger than 5 years of age) tended to select happy faces in response to a variety of odors regardless of valence, whereas older children (5 years of age and older) increasingly adjusted their affective responses according to the pleasantness or unpleasantness of specific smells. Pleasant odors, however, appear to elicit positive affect more robustly across development: pleasant scents enhance positive mood across all ages, including young children, adolescents, and the elderly (Glass and Heuberger, 2016), suggesting that odor-induced positive affect can be robust and less dependent on cognitive maturity. Together, these findings indicate that reliable differentiation of odor valence emerges around 5 years of age, a developmental interval the present design is positioned to examine.

Moral development research points to a comparable developmental period. Empirical evidence is consistent with the view that moral domain differentiation matures gradually rather than being adult-like from the outset. While preschoolers can judge clear violations in both care and purity domains (Curtis and Moore, 2019; Du, 2020), their use of abstract principles, especially for purity norms, remains limited (Danovitch and Bloom, 2009; Smetana et al., 2012). Around 4 to 5 years, children begin to incorporate emotional cues and mental states into their moral evaluations (Wainryb et al., 2005). However, their ability to coordinate multiple contextual cues and apply them selectively across moral domains is still developing. These two developmental lines converge during the preschool years: the capacity to differentiate odor valence emerges around 5 years of age (Cavazzana et al., 2018), while the capacity to integrate emotional and contextual cues into moral evaluation begins to consolidate between 4 and 5 years (Wainryb et al., 2005). The 3-to-6-year age range sampled in Study 2 is therefore positioned to bracket this transition, allowing a comparison between younger preschoolers, in whom both capacities are still immature, and older preschoolers, in whom both have begun to consolidate. Given the immaturity of regulatory processes across this age range, any olfactory influence on moral judgment that does emerge is more likely to be undifferentiated across moral domains than domain-selective.

### The present study

The three frameworks reviewed above generate divergent predictions about how olfactory affect should shape moral judgment across domains and across development, yet the evidence needed to adjudicate among them is lacking. No published study has examined whether ambient odor modulates moral judgment in preschoolers, and few adult studies have crossed odor valence with moral domain in a single design. These gaps matter because the three frameworks make their most divergent predictions when domain and age are crossed: although they may converge on any single condition, they yield distinguishable overall patterns across the full age × domain × valence design.

The present research therefore examined how pleasant, neutral, and unpleasant odors shape moral judgments of care and purity violations in adults (Study 1) and in preschoolers aged 3 to 6 years (Study 2). The 3-to-6-year range was selected to bracket the developmental phase during which odor-valence differentiation and the integration of emotional and contextual cues into moral evaluation both begin to consolidate. The hypotheses are stated at the level of preschoolers as a group rather than at the level of individual year-bands. The year-band analyses describe where within the preschool period any effect is concentrated, and the combined analysis across all four cohorts provides the formal developmental test.

The two samples are reported as separate studies for three reasons. First, the administration procedures were not equivalent: adults read the vignettes themselves, whereas preschoolers heard them read aloud by the experimenter. Treating age as a between-subjects factor in a single pooled model would confound developmental differences with procedural differences. Second, identical items do not guarantee that the rating scale functions equivalently across such distant age groups; the combined analysis reported at the end of Study 2 is therefore exploratory rather than the primary test. Third, Study 1 establishes whether the predicted odor × domain interaction is detectable in adults, whose mature olfactory and cognitive systems provide the most stringent test of the three frameworks; Study 2 then asks when this pattern emerges in early development, the age range at which the frameworks make their most divergent predictions.

Hypothesis 1 (MFT). Unpleasant odors will increase wrongness ratings of purity violations more than of care violations, yielding a significant odor × domain interaction. A strict MFT account predicts this interaction at both ages, provided that children can discriminate odor valence reliably; a Rozin-modified account predicts it more strongly in adults, with weaker or absent selectivity in preschoolers (Rozin et al., 2000).

Hypothesis 2 (Evolutionary and dual-process). Unpleasant odors will increase wrongness ratings in both care and purity domains. The evolutionary account predicts this broad pattern at all ages. The dual-process account predicts it in preschoolers but a care-selective effect in adults, as cognitive control attenuates incidental disgust on rule-based purity judgments.

Hypothesis 3 (Pleasant odor). Pleasant odors will produce more lenient wrongness ratings than neutral odors across both domains and ages, with smaller effects than those of unpleasant odors (Glass and Heuberger, 2016).

The odor × domain interaction in adults provides the key point of divergence among the three hypotheses: stronger effects on purity than care would support H1, comparable effects across domains would support the evolutionary account, and stronger effects on care than purity would support the dual-process account.

## Study 1

### Method

#### Participants

Thirty-six first- to third-year undergraduates (25 female; *M*_age_ = 20.15 years, *SD* = 0.36, range = 19–22) were recruited from universities in Guangzhou, China. The sample size was comparable to or larger than those used in previous experiments examining olfactory effects on moral judgment with within-subjects designs (e.g., Schnall et al., 2008a,b, Study 1, *N* = 40; Wu, 2013, *N* = 32), in which medium-sized effects were reliably detected. All participants completed the three sessions; there was no attrition. No additional demographic data (e.g., socioeconomic status) were collected. Written informed consent was obtained from each participant, and the study was approved by the institutional ethics committee.

### Measures

#### Odor stimuli

Drawing on prior research demonstrating that strawberry scent reliably induces positive affect and fishy odor elicits disgust (Boesveldt et al., 2010; Damjanovic et al., 2018; Leppänen and Hietanen, 2003), three olfactory conditions were employed: pleasant (strawberry essence), unpleasant (fishy odor essence), and neutral (pure water). For each condition, a brown opaque bottle containing 4 mL of the designated solution was placed approximately 30 cm in front of the participant with the cap removed to allow continuous olfactory exposure throughout the task.

#### Care and purity moral judgment vignettes

Moral judgment was assessed using a set of vignettes adapted from established stimulus materials (Clifford et al., 2015; Curtis and Moore, 2019). To facilitate direct comparison with Study 2, only scenarios that were developmentally appropriate and comprehensible for young children were selected. The stimulus set included six scenarios: three representing the care domain and three representing the purity domain. Care scenarios depicted harm to humans or animals (e.g., “You see a person stepping on a kitten’s tail”), whereas purity scenarios involved contamination or hygiene violations (e.g., “You see a person drinking urine”). Following each vignette, participants rated the perceived wrongness of the behavior on a 4-point scale (1 = “not wrong,” 2 = “slightly wrong,” 3 = “very wrong,” 4 = “extremely wrong”). Wrongness ratings within each domain (three care, three purity) were averaged to form care judgment and purity judgment composite scores. Consistent with prior experimental work using vignette-based moral judgment paradigms, these scenarios were treated as experimental stimuli sampling representative violations within each domain rather than as items comprising a psychometric scale; internal consistency estimates are therefore not reported.

### Procedure

**Figure 1:**
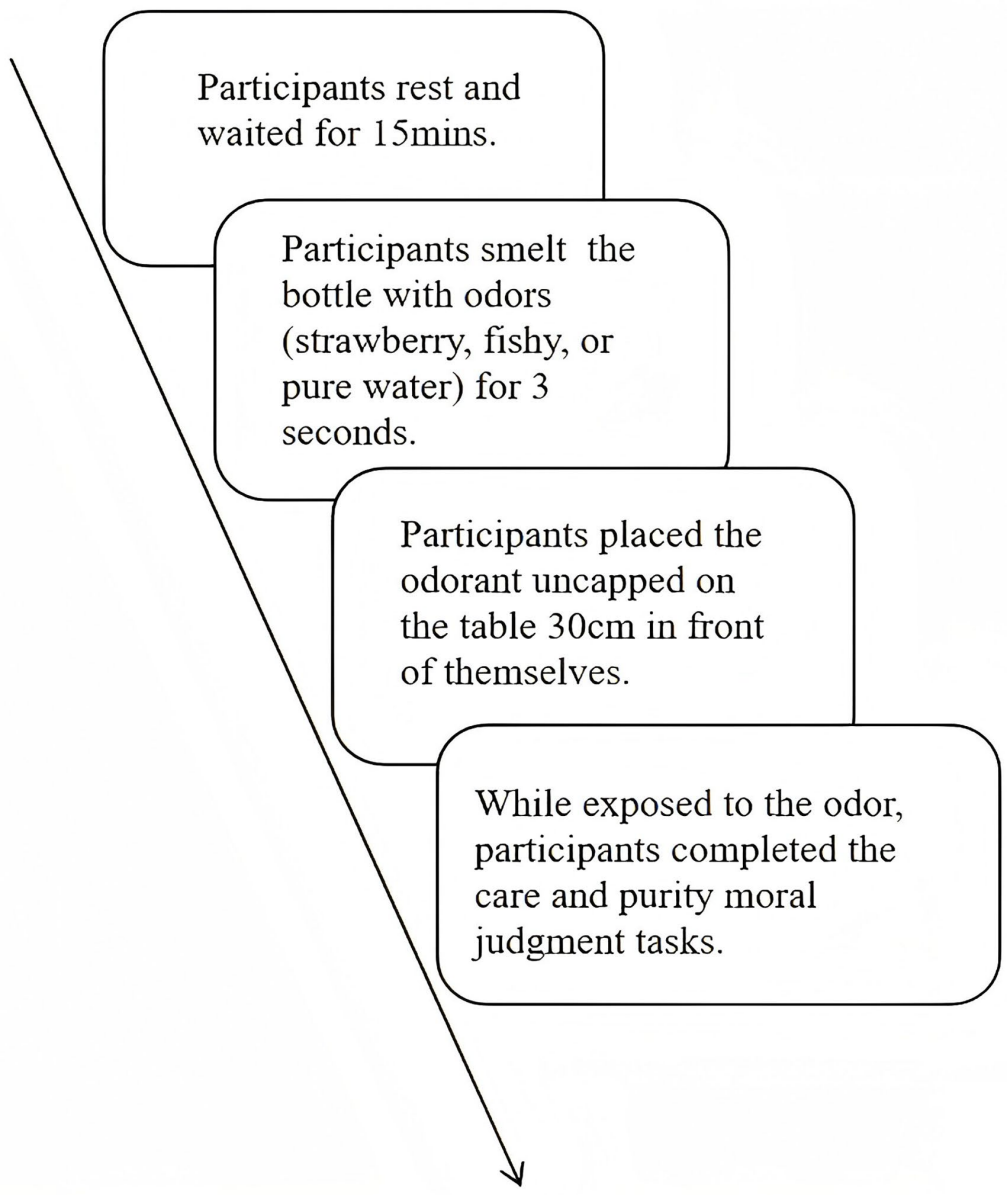
The experiment procedure for adults in Study 1.

We used a within-subjects design. Each participant experienced all three odor conditions and served as their own control. This minimized the influence of individual differences in odor sensitivity and moral baseline. Upon arrival, participants provided informed consent and rested for 15 min in a designated room to stabilize their emotional state before beginning the experiment (Figure 1). Each participant attended three separate sessions, spaced 2 weeks apart to minimize carry-over effects. The same six vignettes (three care, three purity) were presented in each session. Although identical content was used across sessions, the two-week interval was intended to limit episodic memory of prior responses. The order of odor conditions and the sequence of moral scenarios were fully randomized across sessions and participants to control for order and habituation effects. All sessions were scheduled at the same time of day for each participant to reduce circadian variability in mood and olfactory sensitivity.

At the start of each session, participants were instructed to hold a brown bottle containing 4 mL of the assigned odor under their nostrils for 3 s. The bottle was then placed uncapped on the table approximately 30 cm in front of them to maintain continuous olfactory stimulation throughout the task. While exposed to the odor, participants completed all six moral judgment vignettes (three care scenarios and three purity scenarios) in randomized order. The same six vignettes were used in each of the three sessions, with the presentation order re-randomized for each session. All testing was conducted individually in a quiet, isolated room. Each session lasted approximately 10 to 15 min.

After finishing the testing, two manipulation checks were administered. The first check assessed odor-induced affect. Immediately after the moral judgment task, participants reported their current emotional state to verify that the odor successfully induced the intended affective valence. The second check assessed odor preference. Participants rated how much they liked the strawberry odor and how much they disliked the fishy odor on a 3-point scale. These checks were administered after the moral judgment task rather than before it. This timing prevented participants from consciously evaluating the odor before making moral judgments, which would have made the odor cognitively salient and disrupted its function as an incidental affective cue.

### Results

#### Manipulation check

Consistent with previous research showing that strawberry scent induces positive affect while fishy odor elicits disgust (Boesveldt et al., 2010; Damjanovic et al., 2018; Leppänen and Hietanen, 2003), we conducted a manipulation check to assess the effectiveness of the odor priming. Participants self-reported their emotional responses by selecting one of three categorical options (happy, disgusted, or no feeling) after each odor condition. Chi-square goodness-of-fit tests confirmed that the odor manipulations successfully elicited the intended emotional states. Under the fishy odor condition, 28 of 36 participants reported disgust, χ^2^(2, *N* = 36) = 32.17, *p* < 0.001. Under the strawberry odor condition, 26 of 36 reported happiness, χ^2^(2, *N* = 36) = 26.00, *p* < 0.001. Individual differences in odor preference were not associated with moral judgments in the corresponding conditions. Strawberry liking did not correlate with care, *r(34)* = −0.08, *p* = 0.656, or purity judgments, *r(34)* = −0.26, *p* = 0.127. Fishy odor disliking did not correlate with care, *r(34)* = 0.05, *p* = 0.775, or purity judgments, *r(34)* = 0.23, *p* = 0.169. These results indicate that the observed olfactory effects were independent of individual odor preferences.

#### Preliminary analyses

To rule out the possibility that inherent differences between care- and purity-based moral judgments at baseline could confound the observed olfactory effects, we conducted paired-samples *t*-tests comparing wrongness ratings for care and purity violations under the no-odor (control) condition. Wrongness ratings for care violations (*M* = 2.99, *SD* = 0.69) did not significantly differ from those for purity violations (*M* = 2.72, *SD* = 0.82), *t*(35) = 1.78, *p* = 0.084, confirming that the two domains were rated comparably at baseline.

To examine whether gender moderated the olfactory effects on moral judgment, we entered gender as a between-subjects factor in a 2 (domain: care vs. purity) × 3 (odor: pleasant, neutral, unpleasant) × 2 (gender) mixed-design ANOVA. Mauchly’s test indicated a violation of sphericity for the odor factor (*W* = 0.68, *p* = 0.002); the Greenhouse–Geisser correction was therefore applied to all effects involving odor. The main effect of gender was not significant, *F*(1, 34) = 2.17, *p* = 0.150, *ηp*^2^ = 0.06, and gender did not interact with any other factor: gender × odor, *F*(1.51, 52.85) = 1.23, *p* = 0.291, *ηp^2^* = 0.04; gender × domain, *F*(1, 34) = 0.03, *p* = 0.865, *ηp*^2^ = 0.01; gender × odor × domain, *F*(2, 68) = 0.33, *p* = 0.721, *ηp*^2^ = 0.01. Gender was therefore excluded from subsequent analyses.

#### Odor effects on moral judgment

**Figure 2:**
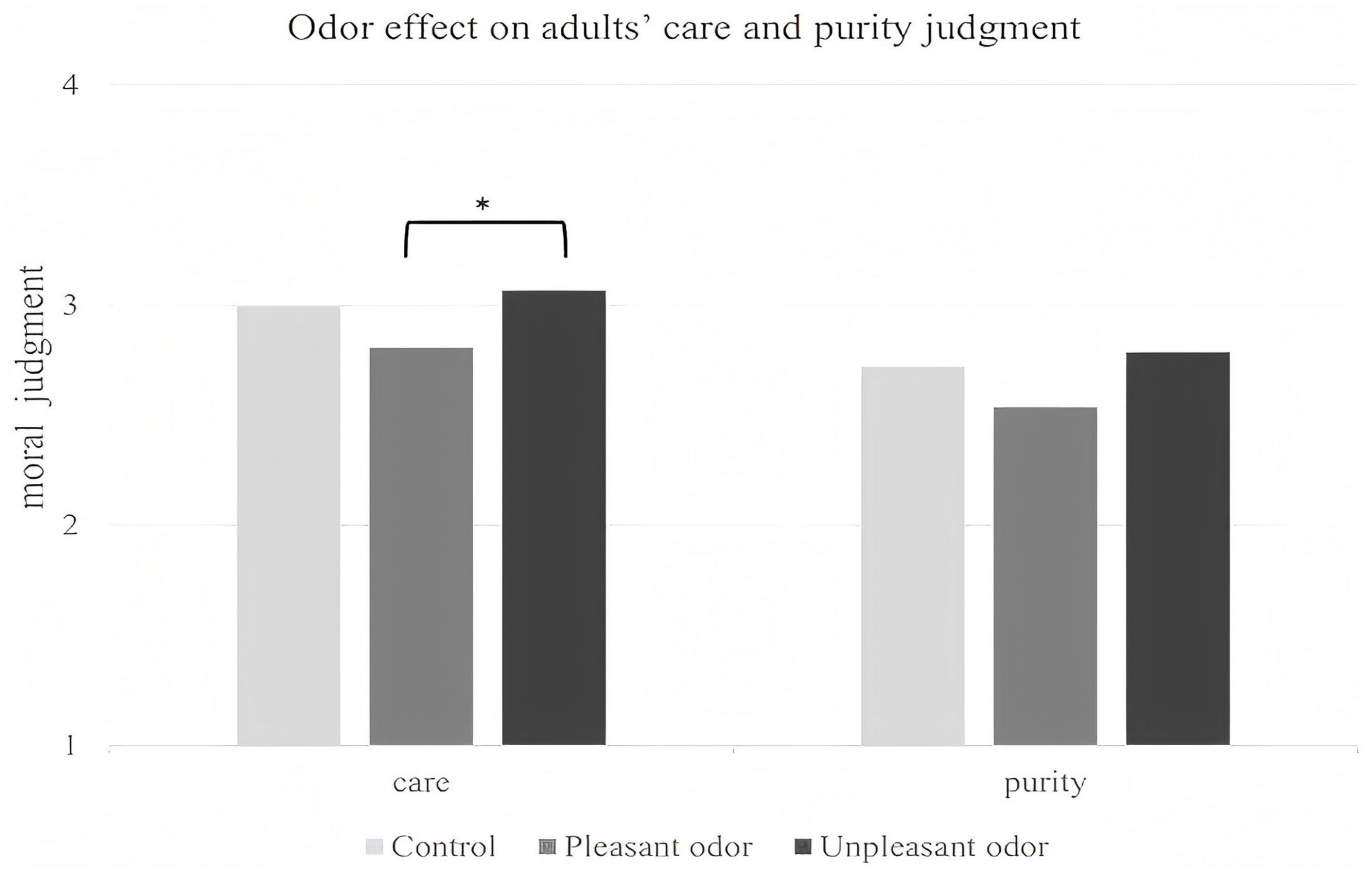
The odor effect on adults’ care and purity judgments. *p < 0.05.

A 2 (domain) × 3 (odor) repeated-measures ANOVA was conducted on the full sample (*N* = 36; see Figure 2). Mauchly’s test indicated a violation of sphericity for the odor factor (*W* = 0.73, *p* = 0.005); the Greenhouse–Geisser correction was therefore applied to the odor main effect. Mauchly’s test was non-significant for the odor × domain interaction (*W* = 0.89, *p* = 0.145), so sphericity-assumed values are reported for that effect. The main effect of domain was not significant, *F*(1, 35) = 1.72, *p* = 0.198, *ηp^2^* = 0.05, indicating that care violations were rated similarly to purity violations. The main effect of odor condition was significant, *F*(1.58, 55.20) = 3.87, *p* = 0.036, *ηp^2^* = 0.10. The domain × odor interaction was significant, *F*(2, 70) = 3.77, *p* = 0.028, *ηp^2^* = 0.10, indicating that the effect of odor differed statistically between the care and purity domains.

#### Simple effects of odor within each domain

Given the theoretically critical nature of domain specificity (see Introduction), we examined odor effects separately within each moral domain using one-way repeated-measures ANOVAs.

For care judgments, Mauchly’s test showed that sphericity was not violated for the odor factor (*W* = 1.00, *p* = 0.938). The main effect of odor condition was significant, *F*(2, 70) = 3.45, *p* = 0.037, *ηp^2^* = 0.09. Pairwise comparisons showed that care violations were judged significantly more harshly under unpleasant odor than under pleasant odor, *t*(35) = 2.48, *p* = 0.018, *d* = 0.41. The pleasant condition was associated with marginally lower wrongness ratings than the neutral condition, *t*(35) = −1.86, *p* = 0.072, *d* = 0.31. The unpleasant condition did not differ significantly from the neutral condition, *t*(35) = 0.74, *p* = 0.465, *d* = 0.12 (Figure 2).

For purity judgments, Mauchly’s test did not indicate a violation of sphericity for the odor factor (*W* = 0.95, *p* = 0.446). The main effect of odor condition on purity judgments was not significant, *F*(2, 70) = 2.12, *p* = 0.128, *ηp*^2^ = 0.06. Pairwise comparisons revealed a marginally significant difference between the unpleasant and pleasant conditions, *t*(35) = 1.86, *p* = 0.071, *d* = 0.31. Neither the unpleasant versus neutral comparison, *t*(35) = 0.58, *p* = 0.566, *d* = 0.10, nor the pleasant versus neutral comparison, *t*(35) = −1.42, *p* = 0.164, *d* = 0.24, reached significance.

#### Study 1 summary

Study 1 characterized the pattern of olfactory modulation of moral judgment in adults. Study 2 extended the paradigm to preschool-aged children (3–6 years) to test whether the pattern observed in adults is already present in early childhood or emerges with the consolidation of domain-differentiated moral judgment.

## Study 2

### Method

#### Participants

Ninety-three preschool children from a public kindergarten in Guangzhou, China, completed all three experimental sessions and were included in the analyses. An additional 42 children were initially enrolled but missed at least one of the three scheduled sessions and were excluded prior to analysis; no further post-enrollment exclusions were applied. Following common practice in the developmental literature, children were grouped by chronological age at first testing into three non-overlapping year bands: 3- to 4-year-olds (3.00–3.99 years; *n* = 26; *M*_age_ = 3.58, *SD* = 0.30; 16 boys), 4- to 5-year-olds (4.00–4.99 years; *n* = 28; *M*_age_ = 4.56, *SD* = 0.29; 13 boys), and 5- to 6-year-olds (5.00–5.99 years; *n* = 39; *M*_age_ = 5.55, *SD* = 0.31; 20 boys). The kindergarten serves families of mixed socioeconomic backgrounds, though no individual-level SES data were collected. Informed consent was obtained from both the kindergarten and the parents before participation.

### Measures

#### Odor stimuli

The same emotional priming protocol as that used in Study 1 was employed, utilizing pleasant, neutral, and unpleasant odor stimuli.

#### Children’s care and purity moral judgment vignettes

Study 2 employed the same set of care and purity vignettes used in Study 1, adapted for use with preschoolers. Scenarios were presented as illustrated stories with simplified language. Given preschoolers’ limited reading ability, all vignettes were read aloud by the experimenter alongside visual aids. Children indicated their judgments using developmentally appropriate response formats (stickers or emoji faces) corresponding to the same 4-point scale used with adults (1 = “not wrong” to 4 = “extremely wrong”). Mean scores were computed separately for each domain by averaging across the three scenarios. As in Study 1, the vignette set served as an experimental manipulation of moral content rather than as a psychometric instrument; the scenarios were drawn from previously validated materials (Clifford et al., 2015) and were designed to represent prototypical violations within each domain.

### Procedure

**Figure 3:**
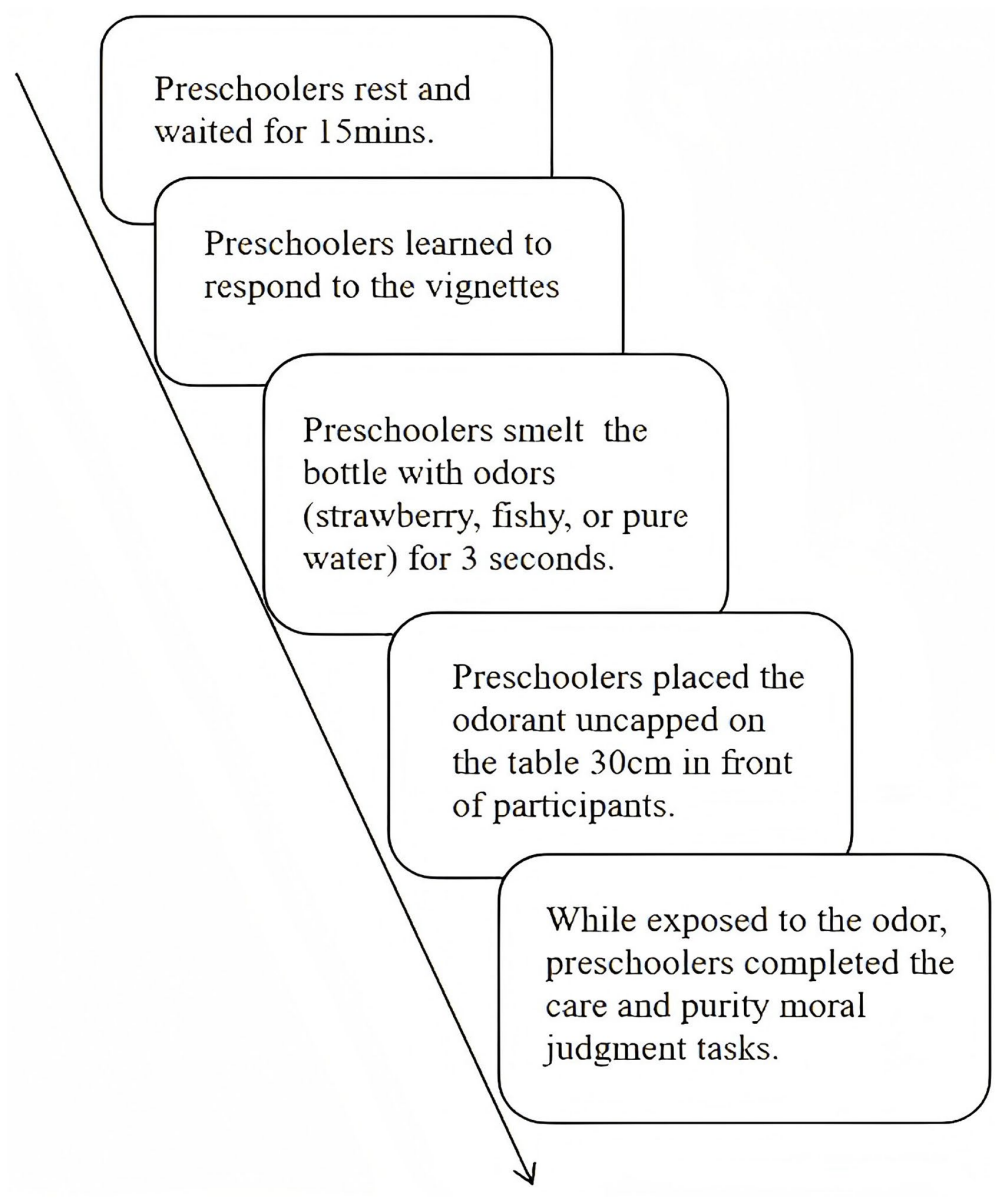
The experiment procedure for preschoolers in Study 2.

A within-subjects design was again adopted so that each child experienced all three odor conditions and served as their own control. The procedure closely mirrored that used with adults in Study 1, with adaptations for developmental appropriateness (Figure 3). Each child attended three separate sessions spaced 2 weeks apart. All sessions were scheduled at the same time of day to reduce circadian variability. The order of odor conditions and the sequence of vignettes were randomized for each child.

All sessions were conducted individually in a quiet room and lasted approximately 15 to 20 min. At the beginning of the first session, children were introduced to the task through simple illustrated practice scenarios to familiarize them with the response format. If a child did not demonstrate comprehension during the initial practice (e.g., could not correctly match the response options to the example scenario), the practice was repeated once. All practice was completed before odor exposure began. For odor presentation, the experimenter held the brown bottle approximately 2 cm below the child’s nostrils for 3 s to ensure adequate initial exposure. The bottle then remained open on the table approximately 30 cm in front of the child throughout the task. Because preschoolers cannot reliably read multi-sentence vignettes, the experimenter read each illustrated scenario aloud while the child viewed the corresponding picture, and the child responded using the practiced format. To minimize experimenter effects, the wording, pacing, and prompts were fully scripted, and the experimenter was blind to the odor condition. The experimenter recorded the child’s responses and noted any behavioral indicators of discomfort or disengagement. After completing all six moral judgment vignettes, children smelled the bottle once more and reported their emotional reaction by selecting one of three categorical options: happy, disgusted, or no feeling. Response cards with corresponding facial expression illustrations were used to support comprehension.

Two manipulation checks were administered at the end of each session. The first check assessed odor-induced affect. Immediately after the final vignette, children reported their emotional state using the categorical response described above to verify that the odor had successfully induced the intended affective valence. The second check assessed odor preference. Children rated how much they liked the strawberry odor and how much they disliked the fishy odor on a 3-point scale. Both checks were administered after the moral judgment task rather than before it. This timing prevented children from consciously evaluating the odor before making moral judgments, which would have made the odor cognitively salient and disrupted its function as an incidental affective cue. All procedures were approved by the institutional ethics committee.

### Results

#### Manipulation check

Chi-square goodness-of-fit tests confirmed that both odor manipulations were effective in the child sample. Under the strawberry odor condition, a significantly greater proportion of children reported feeling happy (79 out of 93, 84.9%) compared to reports of disgust or no feeling, *χ*^2^(2, *N* = 93) = 111.55, *p* < 0.001. Similarly, under the fishy odor condition, reports of disgust (71 out of 93, 76.3%) were significantly more frequent than reports of happiness or no feeling, *χ*^2^(2, *N* = 93) = 77.68, *p* < 0.001. Furthermore, we found that strawberry liking did not significantly correlate with care, *r*(91) = 0.14, *p* = 0.190, or purity judgment, *r*(91) = 0.12, *p* = 0.272; fishy odor disliking was not significantly associated with care, *r*(91) = 0.10, *p* = 0.367, or purity moral judgment, *r*(91) = −0.08, *p* = 0.445. Consistent with Study 1, these results suggest that the observed olfactory effects on children’s moral judgments were independent of individual preferences for the odors themselves.

#### Preliminary analyses

Paired-samples *t*-tests compared wrongness ratings for purity versus care violations in the neutral (no-odor) condition within each age group to verify that children did not differ in baseline evaluations of the two domains. No significant differences emerged in the 3- to 4-year-olds (*n* = 26), *t*(25) = 0.65, *p* = 0.521, 4- to 5-year-olds (*n* = 28), *t*(27) = −1.06, *p* = 0.300, or 5- to 6-year-olds (*n* = 39), *t*(38) = 0.87, *p* = 0.387. Children thus evaluated care and purity transgressions as comparably wrong at baseline, ruling out domain-specific floor or ceiling effects.

A preliminary 3 (odor: pleasant, neutral, unpleasant) × 2 (domain: care, purity) × 2 (gender) mixed-design ANOVA was conducted on the full sample of 93 children. The main effect of gender was not significant, *F*(1, 91) = 0.22, *p* = 0.637, *ηp^2^* = 0.002, and no interaction involving gender reached significance (all *p*s > 0.10). Gender was therefore excluded from all subsequent analyses.

#### Odor effects on moral judgment by age groups

**Figure 4:**
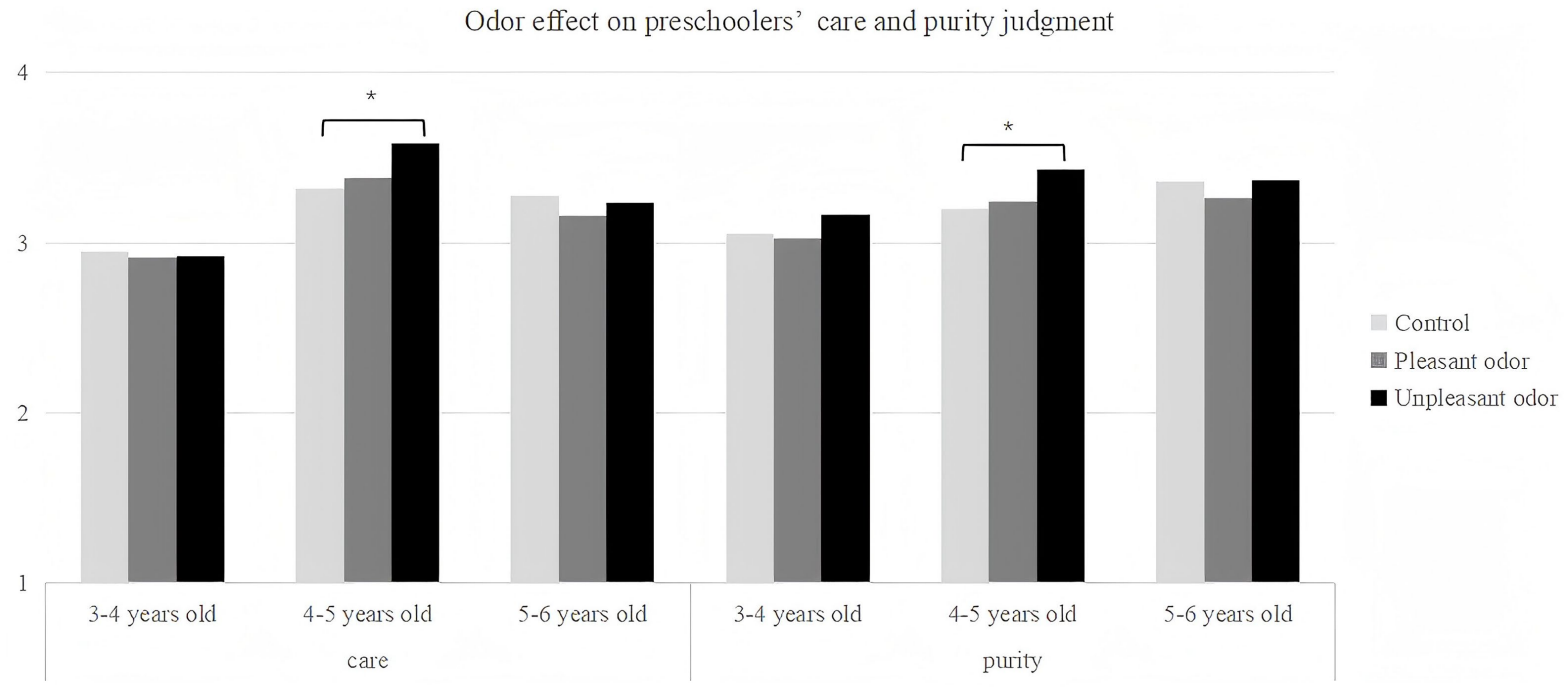
The odor effect on preschoolers’ care and purity judgment. *p < 0.05.

To characterize how olfactory effects on moral judgment were distributed across the preschool age range, we conducted separate 2 (domain) × 3 (odor) repeated-measures ANOVAs within each age group (see Figure 4); a formal test of the developmental contrast is reported in the Combined Analysis below.

Ages 3–4 (*n* = 26). Mauchly’s test indicated that sphericity was tenable for both the odor factor (*W* = 0.93, *p* = 0.408) and the odor × domain interaction (*W* = 0.95, *p* = 0.568). The main effect of odor was not significant, *F*(2, 50) = 0.86, *p* = 0.430, *ηp*^2^ = 0.03. The main effect of domain was not significant, *F*(1, 25) = 0.79, *p* = 0.383, *ηp*^2^ = 0.03. The odor × domain interaction was not significant, *F*(2, 50) = 0.25, *p* = 0.777, *ηp*^2^ = 0.01, indicating that ambient odor did not influence moral judgments in the youngest children. No pairwise comparison was significant in the 3- to 4-year-old group (all *p*s > 0.84).

Ages 4–5 (*n* = 28). Mauchly’s test indicated that sphericity was tenable for both the odor factor (*W* = 0.87, *p* = 0.171) and the odor × domain interaction (*W* = 0.95, *p* = 0.492). The main effect of odor was not significant, *F*(2, 54) = 0.37, *p* = 0.693, *ηp*^2^ = 0.01. The main effect of domain was not significant, *F*(1, 27) = 0.36, *p* = 0.551, *ηp*^2^ = 0.01. The odor × domain interaction was significant, *F*(2, 54) = 4.18, *p* = 0.020, *ηp*^2^ = 0.13, indicating that the effect of odor differed between the care and purity domains in this age group.

To decompose the significant interaction, one-way repeated-measures ANOVAs were conducted for each domain separately. For care judgments, Mauchly’s test indicated that sphericity was tenable (*W* = 0.97, *p* = 0.634). The effect of odor was marginally significant, *F*(2, 54) = 2.92, *p* = 0.062, *ηp*^2^ = 0.10. Because we had directional *a priori* predictions derived from Hypothesis 2, planned comparisons were conducted regardless of the omnibus outcome. Care violations were judged significantly more harshly under unpleasant (*M* = 3.58, *SD* = 0.44) odor than under neutral (*M* = 3.32, *SD* = 0.61), *t*(27) = 2.44, *p* = 0.022, *d* = 0.46. The unpleasant (*M* = 3.58, *SD* = 0.44) versus pleasant (*M* = 3.38, *SD* = 0.58) comparison showed a marginal significance, *t*(27) = 1.86, *p* = 0.074, *d* = 0.35. The pleasant (*M* = 3.38, *SD* = 0.58) versus neutral (*M* = 3.32, *SD* = 0.61) comparison was not significant, *t*(27) = 0.48, *p* = 0.634, *d* = 0.09.

For purity judgments, Mauchly’s test indicated that the sphericity assumption was violated (*W* = 0.79, *p* = 0.043); with the Greenhouse–Geisser estimate, the effect of odor was not significant, *F*(1.65, 44.46) = 2.00, *p* = 0.154, *ηp*^2^ = 0.07. Planned pairwise comparisons nonetheless revealed that purity violations were judged significantly more harshly under unpleasant (*M* = 3.43, *SD* = 0.66) odor than under neutral (*M* = 3.20, *SD* = 0.75), *t*(27) = 2.49, *p* = 0.019, *d* = 0.46. The unpleasant (*M* = 3.43, *SD* = 0.66) versus pleasant (*M* = 3.24, *SD* = 0.61) comparison was not significant, *t*(27) = 1.35, *p* = 0.187, *d* = 0.25. The pleasant (*M* = 3.24, *SD* = 0.61) versus neutral (*M* = 3.20, *SD* = 0.75) comparison was not significant, *t*(27) = 0.28, *p* = 0.782, *d* = 0.05. Taken together, the significant interaction combined with the simple-effects analyses indicates that unpleasant odor elevated wrongness ratings relative to neutral in both care (*p* = 0.022) and purity (*p* = 0.019) domains. Inspection of the condition means revealed that both domains exhibited the same ordinal pattern (unpleasant > pleasant ≈ neutral).

Ages 5–6 (*n* = 39). Mauchly’s test indicated that sphericity was tenable for both the odor factor (*W* = 0.92, *p* = 0.218) and the odor × domain interaction (*W* = 0.90, *p* = 0.151). The main effect of odor was not significant, *F*(2, 76) = 1.45, *p* = 0.240, *ηp*^2^ = 0.04. The main effect of domain was not significant, *F*(1, 38) = 0.55, *p* = 0.464, *ηp*^2^ = 0.01. The odor × domain interaction was not significant, *F*(2, 76) = 1.50, *p* = 0.230, *ηp*^2^ = 0.04. No pairwise comparison was significant in the 5- to 6-year-old group (all *p*s > 0.21).

In summary, substantive interpretation of the developmental pattern, including the absence of effects in the 3- to 4-year-old and 5- to 6-year-old groups, the modest size of each age band, and the implications for theories of moral development are all discussed in the General Discussion. The next analysis combines the adult and child data to test the developmental contrast directly.

#### Combined analysis across age groups

To directly test whether the influence of olfactory cues on moral judgment varies developmentally, data from Study 1 (adults, *N* = 36) and Study 2 (3- to 4-year-olds, *n* = 26; 4- to 5-year-olds, *n* = 28; 5- to 6-year-olds, *n* = 39) were combined into a 3 (odor: pleasant/ neutral/unpleasant) × 2 (domain: care/purity) × 4 (age group: 3- to 4-year-olds/4- to 5-year-olds/5- to 6-year-olds/adults) mixed-design repeated-measures ANOVA. Mauchly’s test indicated a violation of sphericity for the odor factor (*W* = 0.90, *p* = 0.001); Greenhouse–Geisser corrections were applied where appropriate.

The main effect of age group was significant, *F*(3, 125) = 8.91, *p* < 0.001, *ηp*^2^ = 0.176, reflecting systematic differences in baseline moral judgment ratings across development. Neither the main effect of odor nor that of domain reached significance (*ps* > 0.54).

The odor × age group interaction was significant, *F*(5.43, 226.25) = 2.28, *p* = 0.043, *ηp^2^* = 0.052, confirming that the modulatory influence of olfactory cues on moral judgment varies across developmental stages. The odor × domain interaction was also significant, *F*(2, 250) = 4.08, *p* = 0.018, *ηp*^2^ = 0.032, indicating that odors differentially modulated care and purity judgments. The three-way odor × domain × age group interaction did not reach significance, *F*(6, 250) = 1.70, *p* = 0.121, *ηp*^2^ = 0.039, although the moderate effect size and limited observed power (0.642) suggest the analysis may be underpowered to detect higher-order developmental patterns. Together with the within-group analyses reported above, these results support the conclusion that olfactory modulation of moral judgment is reliably observed in adults and the 4- to 5-year-old group but weak or absent in the 3- to 4- and 5- to 6-year-old cohorts.

## General discussion

The present research examined whether ambient odor influences moral judgment and whether such influence depends on moral domain and age. Study 1 tested adults under three odor conditions; Study 2 tested 3- to 6-year-old preschoolers using a developmentally adapted version of the same task; a combined analysis then pooled data across all four age cohorts. Odor effects were detectable in adults and in 4- to 5-year-old children but took different forms in the two groups and were not detectable in the youngest and oldest preschool cohorts.

### Adults: a domain-selective pattern centered on care

The influence of odor on adults’ moral judgment was domain-selective. The odor × domain interaction was reliable: care judgments differed across odor conditions, whereas purity judgments did not. Within the care domain, wrongness ratings were higher under the unpleasant than under the pleasant odor; the pleasant condition produced ratings slightly lower than the neutral condition; the unpleasant–neutral contrast did not reach significance. For purity, the omnibus effect was not reliable, with only a weak trend separating the unpleasant and pleasant conditions.

This pattern does not support Hypothesis 1. Moral Foundations Theory predicts that disgust should selectively intensify condemnation of purity violations (Haidt and Joseph, 2004; Schnall et al., 2008b), yet it was the opposite domain that showed sensitivity. Our finding diverges from early demonstrations of a disgust–purity link (Horberg et al., 2009; Schnall et al., 2008b), but it converges with meta-analytic evidence that incidental disgust effects on moral judgment are small and inconsistent across domains (Landy and Goodwin, 2015), and with later work in which odor manipulations affected non-purity content (Cecchetto et al., 2019; Wu, 2013). The present study extends this line of work in two specific ways. First, it locates the reliable odor effect in the care domain rather than the purity domain, providing a clearer empirical target than the mixed pattern reported in earlier odor studies. Second, it shows that the care-domain effect takes the form of a valence contrast between pleasant and unpleasant odors, rather than a one-sided shift driven only by negative affect. Together, these results argue against treating purity as the privileged target of incidental affect and point toward a broader account in which the affective sensitivity of moral judgment depends on how the judgment itself is computed.

The care-selective pattern is, however, consistent with the adult prediction of Hypothesis 2. Drawing on dual-process theory, H2 predicted that mature executive control could attenuate incidental affect in domains engaging deliberative processing (Greene et al., 2004; Greene and Haidt, 2002). Care scenarios in the present materials depicted concrete, emotionally immediate harm (e.g., stepping on a kitten’s tail), and judgments of such acts are thought to rely heavily on rapid affective appraisal. Incidental affect from an extraneous source is therefore well positioned to shift that appraisal. Purity scenarios depicted contamination acts whose evaluation may engage rule-based reasoning about violations of bodily integrity. In adults with mature executive control, such processing may buffer purity judgments from incidental perturbation. We acknowledge that this interpretation diverges from accounts treating purity judgments as paradigmatically intuitive (e.g., Haidt and Joseph, 2004). Our reasoning is specific to the present materials: for adults with established hygiene norms, the purity scenarios may have been evaluated partly through acquired rules rather than through immediate affective response alone.

An important qualification is that the significant contrast was between the unpleasant and pleasant conditions, not between either and the neutral baseline. We therefore characterize the care-domain finding as a valence contrast rather than a baseline-relative shift. The divergence is nonetheless informative: care judgments tracked ambient affective valence while purity judgments did not.

Baseline wrongness ratings did not differ significantly between domains, although care violations were rated numerically higher. Neither domain approached ceiling on the four-point scale, so both had comparable room for upward modulation. The selective sensitivity of care judgments is therefore unlikely to be an artifact of baseline asymmetry; rather, it suggests that care judgments, grounded in emotionally immediate appraisal, are inherently more permeable to incidental affect.

The modest pleasant-odor effect on care judgments fits this interpretation. Pleasant scents have been shown to promote evaluative leniency and prosocial inclination (Baron and Thomley, 1994; Liljenquist et al., 2010; Glass and Heuberger, 2016). Hypothesis 3 predicted more lenient judgments under pleasant odor, while noting that the affect-as-information framework raises the possibility that positive affect may be less congruent with transgression-focused evaluation (Schwarz and Clore, 1983). The marginal effect, confined to the care domain, provides at best narrow and tentative support for H3.

### Preschoolers: reliable odor effects emerge only at ages 4 to 5

Reliable odor effects emerged only in 4- to 5-year-olds; the youngest cohort showed no detectable effect, and the oldest showed only a non-significant trend resembling the adult ordinal pattern. In this group, the odor × domain interaction was significant, yet planned comparisons showed that unpleasant odor elevated wrongness ratings relative to neutral for both care and purity. Both domains displayed the same ordinal pattern (unpleasant > pleasant ≈ neutral), and the two mean shifts were nearly identical. The significant interaction likely reflected differences in response variability across conditions, not a real difference in how odor affected care versus purity judgments.

These effects must be interpreted cautiously. The omnibus ANOVA reached only marginal significance for care and did not reach significance for purity. The significant pairwise comparisons were obtained through planned contrasts motivated by directional *a priori* predictions. Within each domain, only the unpleasant versus neutral contrast was reliable; the unpleasant versus pleasant comparison did not reach significance, and the pleasant versus neutral comparison was non-significant throughout. The subsample was too small to provide adequate statistical power. The findings should therefore be treated as preliminary evidence of a bounded developmental phase, not as a firmly established effect.

That unpleasant odor elevated wrongness ratings in both domains, rather than selectively in purity, is consistent with H2’s preschool prediction: broad, undifferentiated olfactory modulation reflecting limited cognitive regulation. It is inconsistent with H1’s prediction that disgust should selectively amplify purity condemnation.

We propose two converging conditions for the narrow developmental phase. First, the odor must register as differentially negative. Children below approximately 4 years tend to assign positive or undifferentiated affect to odors regardless of objective valence (Cavazzana et al., 2018; Sorokowska et al., 2015), so the fishy odor may not have generated a reliably negative input for our 3- to 4-year-olds. Second, the resulting affect must reach moral evaluation without being filtered. By 5 to 6 years, regulatory and source-monitoring abilities begin to mature (Smetana et al., 2012; Wainryb et al., 2005), allowing children to discount irrelevant affect. Between these boundaries, at 4 to 5 years, the odor is perceived as unpleasant but not yet filtered, producing broad elevation across both domains. This account is *post hoc* and rests on cross-sectional comparisons with modest subsample sizes.

The absence of reliable effects in the oldest cohort calls for explanation. Two complementary explanations are possible. First, cognitive control may already be dampening the broad odor effect seen in younger preschoolers. However, the adult-like domain-selective pattern has not yet formed. The result is a gap where neither pattern is clearly detectable. Second, the modest subsample size may have been insufficient to detect an attenuated effect; the descriptive means followed the adult ordinal pattern, consistent with an emerging but underpowered trend. Both explanations are speculative.

The absence of pleasant-odor effects across all preschool cohorts is consistent with the affect-as-information account: negative affect is congruent with transgression-focused evaluation, whereas positive affect is not, reducing its capacity to shift moral judgments, particularly in preschoolers whose capacity to integrate subtle affective cues is still developing (Schwarz and Clore, 1983).

### Olfactory modulation varies across development

The combined analysis directly addressed the central developmental question. Pooling all age cohorts yielded a significant odor × age group interaction, confirming that olfactory modulation of moral judgment is not constant across development. The odor × domain interaction was also significant, indicating that odors did not act equivalently on care and purity content across the full sample. The three-way odor × domain × age group interaction did not reach significance and was likely underpowered. We therefore stop short of claiming that the adult pattern (care-selectivity) and the 4- to 5-year-old pattern (relative domain generality) constitute a statistically reliable developmental dissociation. The data support a narrower conclusion: olfactory modulation changes across development. Whether its scope narrows from broad to selective with age requires confirmation in larger and longitudinal samples.

Although the three-way interaction was not reliable, the contrast structures differed descriptively across age groups. In adults, the significant contrast in the care domain was between unpleasant and pleasant conditions, reflecting a bidirectional valence divergence. In 4- to 5-year-olds, the significant contrast was between unpleasant and neutral, indicating a baseline-relative elevation. The adult pattern is consistent with a tonic affective context in which opposing valences pull care judgments apart; the preschool pattern is consistent with a more direct injection of negative affect into an evaluative system that lacks the regulatory capacity to discount it.

Across the two studies, the developmental pattern is most consistent with the dual-process framework. At the domain level, dual-process theory predicts that judgments relying on rapid affective appraisal will be more susceptible to incidental affect than those engaging deliberative processing (Greene et al., 2004; Greene and Haidt, 2002). The adult data support this: care judgments were sensitive to olfactory valence contrast while purity judgments were not. At the developmental level, the same framework predicts that young children, whose executive control is still immature, will show broader and less domain-differentiated effects (Smetana et al., 2012; Wainryb et al., 2005). The 4- to 5-year-old data support this: unpleasant odor elevated wrongness ratings in both domains. The trajectory from broad modulation in young children to domain-selective modulation in adults thus reflects the progressive maturation of regulatory processes rather than the unfolding of an innate emotion–domain architecture.

By contrast, the MFT prediction was not supported at either developmental endpoint. In adults, care rather than purity showed olfactory sensitivity. In preschoolers, the youngest cohort to show reliable effects displayed a broad rather than purity-selective pattern. An MFT proponent might counter that the disgust–purity coupling requires full maturation of disgust as a differentiated emotion. Even granting this, purity-selective modulation should emerge alongside or shortly after the first detectable olfactory effect on moral judgment; the broad 4- to 5-year-old pattern does not fit this expectation. The absence of purity-selectivity at that earliest point is more naturally accommodated by the dual-process account, on which domain-selective patterns emerge only as cognitive control matures sufficiently to filter incidental affect in some contexts but not others (Greene et al., 2004). The evolutionary olfactory hypothesis correctly predicted broad effects but did not anticipate adult domain-selective narrowing. Of the three frameworks considered, the dual-process model most readily accommodates both ends of the developmental spectrum and provides a mechanistic account of the transition between them.

Hypothesis 3 received at most marginal support. In adults, the pleasant-odor effect was confined to the care domain and reached only marginal significance. In preschoolers, pleasant odor produced no detectable shift. The asymmetry between unpleasant and pleasant effects is consistent with the affect-as-information account: negative affect is congruent with transgression-focused evaluation, whereas positive affect is not (Schwarz and Clore, 1983).

### Contribution and limitations

The present work contributes to the literature in three ways. First, it extends odor-based moral modulation research into the preschool years and identifies a narrow developmental window at 4–5 years during which ambient odor shifted moral evaluation. Second, the combined analysis provides direct statistical evidence, through the significant odor × age group interaction, that olfactory modulation of moral judgment is developmentally variable: 4- to 5-year-olds showed broad elevation under unpleasant odor, whereas adults showed a care-selective valence contrast. This shift does not fit the nativist prediction that a disgust–purity coupling should be present at the earliest age at which any olfactory effect emerges (Haidt and Joseph, 2004). It fits more naturally with the dual-process account in which mature cognitive control differentially buffers moral domains from incidental affect (Greene et al., 2004; Smetana et al., 2012). Third, the adult finding of selective care-domain sensitivity contributes to the unresolved debate about the scope of incidental disgust effects (Landy and Goodwin, 2015), suggesting that adult care judgments are more permeable to incidental affect than purity judgments are.

Several limitations bear on these conclusions, though three design features constrain how strongly they undermine the central claims: the within-subjects domain manipulation, the use of identical scenarios across age groups, and the pooling of all four cohorts in the combined analysis. The principal limitation is sample size: no cohort reached 80% power for the observed effect magnitudes, and the three-way interaction was likewise underpowered. We therefore frame the 4- to 5-year-old finding as preliminary. We do not claim a statistically reliable three-way developmental dissociation. Three contrasts did, however, survive conventional thresholds. Adults showed care-selective sensitivity. The 4- to 5-year-old group showed broad elevation across both domains. The modulation of odor effects changed reliably with age. Although moral wrongness ratings used continuous scales for both adults and children, the manipulation check for odor perception relied on a categorical emotion-selection task and a three-point preference rating. A continuous check would have captured individual differences in odor-evoked affect more precisely, since the same odorant can elicit somewhat different responses across individuals; we retained the simpler format to avoid drawing attention to the odor as a task-relevant cue and inducing demand characteristics, and the categorical check did confirm the intended valence direction in every cohort. Each valence category was represented by a single odorant, which limits generalization to pleasant or unpleasant odors more broadly; testing multiple exemplars would have required additional sessions to avoid carry-over and olfactory adaptation, which was not practical with preschoolers given the existing three-session protocol. The two studies also differed in delivery format: adults read vignettes themselves, whereas children heard them read aloud by a blind, scripted experimenter. This difference was unavoidable given the children’s reading limits, but it remains a source of variation that the design could not fully remove. The task included no morally neutral fillers, the same vignettes were reused across sessions with a two-week interval, and the adult sample was predominantly female. Family socioeconomic status, parental education, and moral socialization practices were not measured; these variables are unlikely to drive within-session odor × domain interactions but may shape baseline moral severity. Not every methodological consideration can be addressed in a single experiment, and future work should employ larger and longitudinal samples, multiple odorants per valence category across counterbalanced sessions, parallel vignette pools, and family-environment covariates.

## Conclusion

Ambient odor can shape how people judge moral transgressions, but its influence is neither uniform across moral domains nor stable across age. The results suggest that this influence first appears between ages 4 and 5. At this age, children can tell pleasant from unpleasant smells, but they cannot yet set aside task-irrelevant emotional responses. The effect then narrows with age. As cognitive control matures, children gain the ability to shield specific moral domains from incidental affect. Rather than reflecting a fixed linkage between disgust and a specific moral domain, olfactory modulation of moral evaluation appears to be a developmentally dynamic phenomenon shaped by the interplay between affective input and the regulatory resources available to the judge.

## Data Availability

The raw data supporting the conclusions of this article will be made available by the authors, without undue reservation.
